# Facial soft tissue changes and volumetric analysis of upper airways in patients undergoing surgically assisted rapid maxillary expansion using a transpalatal distractor^☆^

**DOI:** 10.1016/j.bjorl.2023.101372

**Published:** 2023-12-18

**Authors:** Carlos Augusto de Jesus Oliveira Gonçalves, João de Jesus Viana Pinheiro, Marcelo Newton Carneiro, Ana Karla da Silva Tabosa, Roberto Carlos Rivadeneira Cárdenas, José Thiers Carneiro

**Affiliations:** a Hospital Ofir Loyola, Belém, PA, Brazil; b Universidade Federal do Pará, Programa de Pós-Graduação em Odontologia, Belém, PA, Brazil; c Universidade Federal do Pará, Belém, PA, Brazil

**Keywords:** Maxillary expansion, Soft tissue changes, Transpalatal distractor, Airway

## Abstract

**Objective:**

To verify changes in facial soft tissue using the RadiANT-DICOM-viewer and Dolphin Imaging software, through linear measurements of tomographic points in a 3D reconstruction of the face and volumetric evaluation with three-dimensional measurements of the upper airways of patients with transverse maxillary discrepancy undergoing Surgically Assisted Rapid Maxillary Expansion (SARME).

**Methods:**

Retrospective, transverse, and descriptive study, through the analysis of computed tomography scans of the face of patients with transverse maxillary discrepancy, treated from July 2019 to December 2022. The sample consisted of 15 patients of both sexes, aged 21–42 years old, who underwent surgically assisted rapid maxillary expansion using the transpalatal distractor. Analysis was performed through linear, angular, and three-dimensional measurements in millimeters, in the preoperative and late four-month postoperative period, in frontal 3D tomographic images of the face, in the region of the width of the nose and alar base and also angular measurement in the lateral tomography for the angle nasolabial and upper airways of rhinopharynx, oropharynx and hypopharynx.

**Results:**

There was an increase in nasal width with an average of 1.3467 mm and an increase in the alar base with an average of 1.7333 mm. A significant difference was found in the pre- and postoperative assessments of the measurements of nasal width, alar base and nasolabial angle, as well as the upper airways in all their extension. The results favour a better understanding of the professional and the patient regarding the diagnosis and management of patients with transverse maxillary width discrepancies.

**Conclusion:**

Although our study shows an increase in soft tissues after SARME, no aesthetic changes are observed clinically, and all patients report significant respiratory improvement. SARME may therefore contribute to the improvement of professionals working in the field of oral and maxillofacial surgery and orthodontics.

**Level of evidence:**

Level 4.

## Highlights

• Changes were observed in the angular measurements (nasolabial angle).
• Changes were observed in the facial soft tissue (nasal width and alar base).
• Airway volumetric increase after SARME.

## Introduction

Maxillary atresia is considered a form of skeletal deformity characterized by a discrepancy in the maxilla/mandible relation in the transverse plane.^1^, ^2^ Surgically Assisted Rapid Maxillary Expansion (SARME) is a surgical procedure for the correction of transverse maxillary deficiency.^3^, ^4^, ^5^, ^6^ As characteristics of this deformity, patients have a posterior crossbite, which can be unilateral or bilateral, an ogival-shaped palate, loss of parabolic conformation of the dental arch (“V-shape”) and dental crowding.^3^, ^4^, ^7^ The main indication of SARME is the increase in the transverse width of the atretic maxilla, but effects are seen in the entire nasomaxillary complex, such as soft tissues and airways.^1^, ^2^, ^3^, ^6^

There are several devices that can be used for maxillary expansion, such as dental anchorage devices; the Haas and Hyrax are the most used and widespread, in addition to bone anchorage distractors.^6^, ^7^, ^8^ Distractors such as dental anchorage devices can present numerous complications during bone expansion, such as excessive buccal torque of the teeth, periodontal defect on the buccal surface of the expanding teeth and tissue necrosis of the palate, depending on whether or not acrylic is used. On the other hand, distractors with bone anchorage eliminate these intercurrences, as well as bone expansion without buccal torque on the teeth.^3^, ^9^, ^10^, ^11^, ^12^

Bone and dental responses after SARME have been widely investigated.^13^ Although there is little evidence surrounding permanent changes in the patient’s facial profile and airway after SARME, it has been suggested that changes in hard tissue are not always followed by overlying soft tissue.^9^, ^12^

Most authors have investigated the effects of soft tissue changes through teleradiographs. Direct measurements or frontal photographic analysis were proposed but proved to be limited. Lateral cephalometric radiographs and photographs provided two-dimensional (2D) data that do not allow three-dimensional (3D) analysis. In addition, direct measurements on the face are necessarily accompanied by soft tissue distortion or the identification of difficult points.^6^, ^12^, ^13^

Due to the inaccuracy of these methods to quantify soft tissue changes, the proposal of alternative techniques has increased. An example of this is 3D analysis through computed tomography scans of the face.^6^, ^13^

Three-dimensional measurements proved to be more accurate than linear ones. Extraoral scanners recently introduced in the dental and orthodontic field could represent a good alternative to previous proposals to better understand the relationships between soft tissue and hard tissue before and after surgical and orthodontic treatment.^6^, ^12^

The aim of this study was to evaluate the dimensional changes in the facial soft tissue, through measurements of 3D and 2D tomographic points and volumetric alterations of the upper airways, using multislice computed tomography-scans (CT-scans) of patients with transverse maxillary discrepancy submitted to SARME.

## Methods

This retrospective study was carried out by the Oral and Maxillofacial Surgery team at Ophir Loyola Hospital. Based on previous studies, the required sample to carry out this study was calculated to be 16 patients. The sample number obtained was 15 patients for soft tissue analysis, 2 of which were discarded due to having incomplete tomographic examinations; the final sample number was 13 patients, 7 women and 6 men, with a mean age of 32.2 (21–51) years old, with a power of 0.73 and 15 for airway assessment. Thirty CT-scans were used: 15 preoperative CT scans and 15 late postoperative CT scans at 4 months.

The study was approved by the Human Research Ethics Committee of the of the Ophir Loyola Hospital, Belém do Pará – CAAE: 62591822.3.0000.5550. Appraisal 5,702,295.

The patients were operated on by the same surgeon; all patients had maxillary atresia and underwent a surgical procedure in which bilateral mucoperiosteal incisions and detachment were performed, made from the piriform edges to the zygomatic buttress, as well as bilateral Le Fort I osteotomies, from the piriform borders to the pterygomaxillary junction. The sagittal osteotomy of the maxilla was performed using a delicate osteotome between the central incisors, parallel to the palate, obtaining a space of approximately 1–1.5 mm. A suture was performed with resorbable material (2.0 vycril) on the alar base suture and 4.0 vycril in the buccal maxillary access region with a V–Y suture.

A transpalatal distractor (Rapid Palatal Expander, KLS Martin, KG. Germany®) was fixed in the region of the first molars using a screw on each side. The distractor was activated until separation was noticed between the central incisor teeth. Mobilization was continued until there was approximately 1.5–2 mm between the central incisors. Subsequently, the device was closed and activated in the postoperative period after one week. Activation followed the manufacturer's instructions, which recommends 0.33 to 0.66 mm per day (1–2 coloured markings).

Computed tomography scans were performed at two different times: before SARME (T1) and after 4 months of device use (T2).

3D images were processed from DICOM (Digital Imaging and Communications in Medicine) and constructed and aligned with the help of the software Radiant-DICOM-Viewer (64-bit; Poznan, Poland). The analysis offers the possibility to obtain multiplanes slices (sagittal, axial, and coronal) of the CT images. Preoperative and four-month late postoperative linear measurements were taken.

The images were oriented in the Frankfurt horizontal planes, the coronal planes of the labial commissure parallel to the lower edge of the orbit and the sagittal planes. To assess soft tissue changes, 3D cephalometric analyses were performed with specific linear points listed in Table 1. The analyses were performed by the same calibrated examiner. The width of the soft tissue of the nose was measured in 3D reconstruction at two different points: first, measuring the linear distance (mm) between points Alar (Al) AlE and AlD (nasal width) and second, measuring the distance between points Alar curvature (Ac) AcE and AcD (width of the alar base) in the pre- and postoperative period.

**Table 1:** Points located in the soft tissue.

Lip filter (Lf)	Vertical depression in the middle area of ​​the upper lip
Pronasal (Prn)	The most prominent point on the nose located in the midline
Alar (Al)	The most lateral point on the outer side of each nostril
Alar curvature (AC)	Point located at the soft tissue insertion of each alar base
Subnasal (Sn)	The midpoint between the junction of the inferior border of the nasal septum and the upper lip, in the midline

Angular measurements on soft tissue sagittal tomographic images were used to assess the nasolabial angle formed by the pronasal (Prn), subnasal (Sn) and philtrum points in patients pre- and postoperatively.

**Figure 1:**
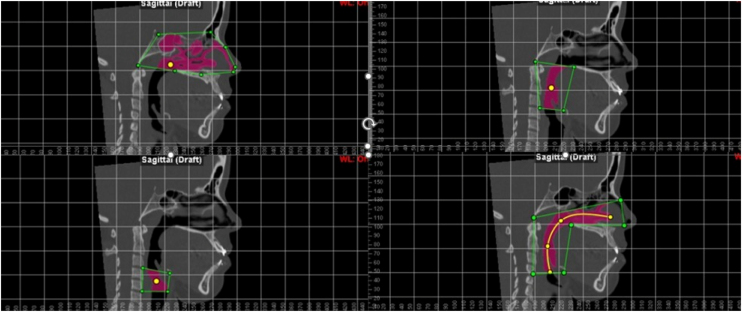
Boundaries of the nasopharynx, oropharynx and hypopharynx in the software.

For the evaluation of the upper airways, the Dolphin Imaging software (Version 11.95.08.73 Premium, 2021 Chatsworth CA, USA) was used. The images were oriented in the Frankfurt horizontal planes, the coronal planes of the labial commissure parallel to the lower edge of the orbit and the sagittal planes. The airway segmentation process was systematized as follows: A marker for defining the intersection region, referred to in the software as “seed point”, the anatomical limits of each sub-region were established by the authors.^14^ Subsequently, the boundaries of the nasopharynx, oropharynx and hypopharynx were contoured within each separate region in a two-dimensional take, after which the software calculated the total airway in volume (mm^3^) (Fig. 1).^14^

### Statistical analysis

Statistical analysis was performed using the Bioestat 5.3 software (Institute for Sustainable Development Mamirauá, Belém do Pará, Brazil).

Normality was checked using the Shapiro–Wilk test, using Student’s t-test to compare two quantitative and dependent normal distributions in two groups. One preoperative and one postoperative assessment was performed with the Student's t-test for normal distributions and Wilcoxon test for abnormal distributions. A p-value less than 0.05 was considered significant when testing differences between measurements taken pre- and post-SARME.

## Results

**Figure 2:**
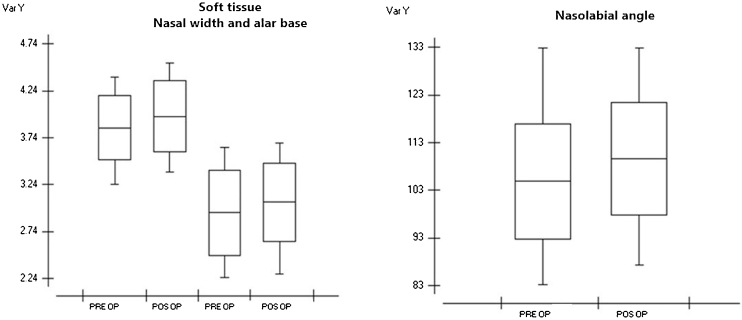
Boxplot showing pre- and post-operative measurements of nasal width, alar base, and nasolabial angle. Graph 1 — Showing the difference between pre- and postoperative means of the nasal width preoperative means of 38.43 mm and postoperative values, we obtained an average of 39.62, and Alar Base groups we obtained a mean preoperative value of 29.3 mm and postoperative values we obtained an average of 30.5 mm. Graph 2 — Showing pre- and post-operative angle measurements we obtained an average of 104.6° in preoperative data and in the postoperative evaluation we obtained an average of 109.5°.

In the measurements made in the preoperative data regarding nasal width, we obtained an average of 38.43 mm with a standard deviation of 0.343 mm. In the postoperative values, we obtained a mean of 39.62 mm and a standard deviation of 0.378 mm; the smallest difference was 0.2 mm, and the largest difference was 3 mm, with a mean difference of 1.37 mm. We obtained a p-value of 0.0007 after statistical evaluation using Student's t-test, showing that there was a significant difference between the groups (Table 2). In the alar base measurements, we obtained a mean preoperative value of 29.3 mm and a standard deviation of 0.456 mm. In the postoperative values, we obtained a mean of 30.5 mm and a standard deviation of 0.417 mm. The smallest difference was −1.5 mm and the largest difference was 2.8 mm, with a mean difference of 1.54 mm. A p-value of 0.0030 was obtained after statistical analysis using Student's t-test (Table 3), which showed that there was a significant difference between the groups (Fig. 2).

**Table 2:** Facial soft tissue and nasolabial angle measurements.

	Nasal width			Wing base			Nasolabial angle		
	Pre-OP	Pos-OP	DIF	Pre-OP	Pos-OP	DIF	Pre-OP	Pos-OP	DIF
PAT 01	43.8	45.3	1.5	33.7	35.9	2.2	103.6	113.1	9.5
PAT 02	41.1	44.1	3	29.7	31.2	1.5	103.8	111.8	8
PAT 03	42	42.8	0.8	33.7	34.6	0.9	82.9	95.2	12.3
PAT 04	41.1	41.7	0.6	28	29.1	1.1	100.7	104.8	4.1
PAT 05	36.1	36.4	0.3	29.4	30.7	1.3	106.7	109.1	2.4
PAT 06	41.1	43.4	2.3	25.2	28	2.8	99.5	110.5	11
PAT 07	32.4	33.7	1.3	22.4	22.8	0.4	95.7	97.4	1.7
PAT 08	38.7	39.6	0.9	31.3	31.8	0.5	106.4	112.9	6.5
PAT 09	36.2	37.9	1.7	24.1	26.6	2.5	93.2	87.2	−6
PAT 10	33	34.3	1.3	22.7	24.9	2.2	104.7	109.4	4.7
PAT 11	37.6	37.8	0.2	32.2	33	0.8	132.8	132.8	0
PAT 12	37.4	37	0.4	32.9	31.4	−1.5	116.4	116.9	0.5
PAT 13	39.1	41.6	2.5	36.3	36.8	0.5	114.5	122.4	7.9
Average	38.43	39.62	1.37	29.35	30.52	1.17	104.68	109.50	5.1
SD	0.343	0.378		0.4564	0.4173		7.397	8.889	

The p-value was = 0.0007 for nasal width groups and = 0.0030 for alar base; the p-value was 0.0053 for the nasolabial angle group.

PAT, patient; SD, standard deviation; OP, operative; DIF, difference.

**Table 3:** Nasopharynx, oropharynx, hypopharynx and total airway measurements.

	NASO			ORO			HYPO			Total		
+	Pre-OP	Pos-OP	DIF	Pre-OP	Pos-OP^a^	DIF	Pre-OP	Pos-OP	DIF	Pre-OP	Pos-OP	DIF
PAT 01	65,025	66,258	1233	15,469	15,567	98	4435	4552	117	91,723	105,863	14,140
PAT 02	44,253	55,324	11,071	8727	10,243	1516	1763	3103	1340	57,175	74,128	16,953
PAT 03	71,878	74,810	2932	9727	10,678	951	1867	4006	2139	50,129	61,465	11,336
PAT 04	73,758	83,166	9408	10,556	13,184	2628	2974	3208	234	78,226	84,701	6475
PAT 05	70,265	77,658	7393	14,420	20,174	5754	4055	5082	1027	90,045	106,478	634
PAT 06	54,161	57,985	3824	12,460	29,575	17,115	4782	8760	3978	105,844	134,616	28,772
PAT 07	17,610	65,704	48,094	16,873	71,909	55,036	3657	3823	166	87,461	93,310	5849
PAT 08	60,947	61,668	741	6911	7162	251	1098	2461	1363	56,039	67,780	11,741
PAT 09	101,290	106,316	5026	12,902	16,553	3651	8042	8418	376	122,609	125,337	2728
PAT 10	67,509	69,556	2047	3975	4244	269	1114	1252	138	53,678	58,487	4809
PAT 11	52,795	59,747	6952	8518	13,680	5162	4100	4522	422	55,667	55,673	6
PAT 12	40,935	45,038	4103	7079	10,384	3305	684	772	38	47,439	55,190	7751
PAT 13	43,932	47,089	3157	13,357	24,074	10,717	4536	5274	738	69,513	85,877	16,364
PAT 14	60,308	76,926	16,618	16,802	28,337	11,535	3878	3890	17	82,640	83,058	418
PAT 15	16,702	29,276	12,574	9717	13,203	3486	3098	4079	990	72,560	79,084	6524
Average	56,929	64,263	7334	11,166		8089	3348	4204	856	74,716	84,736	10,020
SD	20,315	20,149		3844			1881	2196		22,146	24,659	4978
Median					13,680*							

The p-value was = 0.0451 for nasopharynx groups,  = 0.0401 for oropharynx,  = 0.0007 for hypopharynx and p < 0.0001 for total airway group.

PAY, patient; SD, standard deviation; OP, operative; DIF, difference; NASO, nasopharynx; ORO, oropharynx; HYPO, hypopharynx; total, total airway.

a ORO abnormal data.

Evaluating the nasolabial angle, we obtained a mean of 104.6° with a standard deviation of 12.120 in the preoperative data. In the postoperative evaluation, we obtained a mean of 109.5° with a standard deviation of 11.780; the smallest difference was −6° and the largest difference was 12.3°. We obtained a p-value of 0.0053 after statistical analysis (Table 2), demonstrating that there was a significant difference between the groups (Fig. 2).

**Figure 3:**
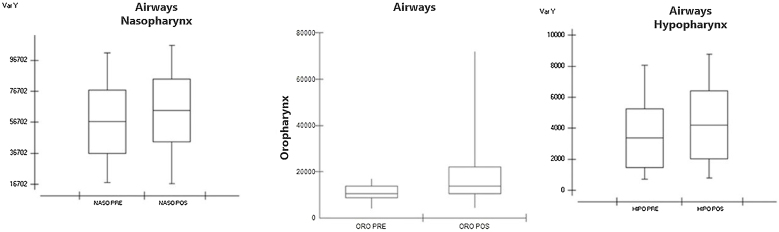
Boxplot showing pre- and post-operative measurements of nasopharynx, oropharynx, and hypopharynx. Graph 3 — In the measurements taken in the pre-operative data referring to the nasopharynx we obtained an average of 56.929 mm^3^ and in the post-operative values we obtained an average of 64.263 mm^3^. Graph 4 — In oropharynx measurements we obtained a preoperative median of 10,556 mm^3^ and interquartile deviation of 5266 mm^3^ and in postoperative values we obtained a median of 13,680 mm^3^. Graph 5 — In the hypopharynx, we obtained an average of 3348 mm^3^ preoperatively and an average of 4204 mm^3^ postoperatively.

In the measurements made in the preoperative data referring to the nasopharynx, we obtained an average of 56,929 mm^3^ with a standard deviation of 20,315 mm^3^. In the postoperative values, we obtained a mean of 64.263 mm^3^ and a standard deviation of 20.149 mm^3^. The smallest difference was 741 mm^3^ and the largest difference was 48,094 mm^3^. We obtained a p-value of 0.0451 after statistical evaluation using Student's t-test (Table 3), which showed that there was a significant difference between the groups (Fig. 3). In the oropharynx measurements, we obtained a preoperative median of 10,556 mm^3^ and an interquartile deviation of 5266 mm^3^; in the postoperative values we obtained a median of 13,680 mm^3^ and interquartile deviation of 11,593 mm^3^, with p-value = 0.0007 after statistical analysis using the Wilcoxon test (Table 3), showing that there was a significant difference between the groups (Fig. 3).

**Figure 4:**
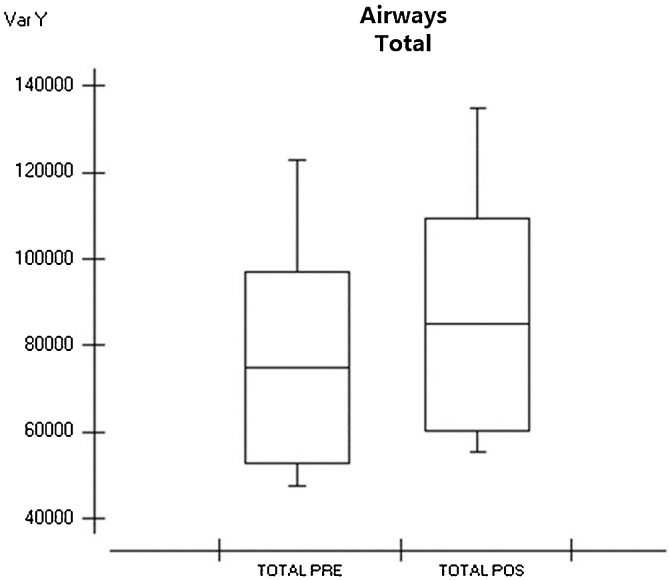
Boxplot showing pre- and postoperative airway measurements total. Graph 6 — Evaluating the total airway, we obtained an average of 3348 mm^3^ in preoperative data and in the postoperative evaluation we obtained an average of 74,716 mm^3^.

**Figure 5:**
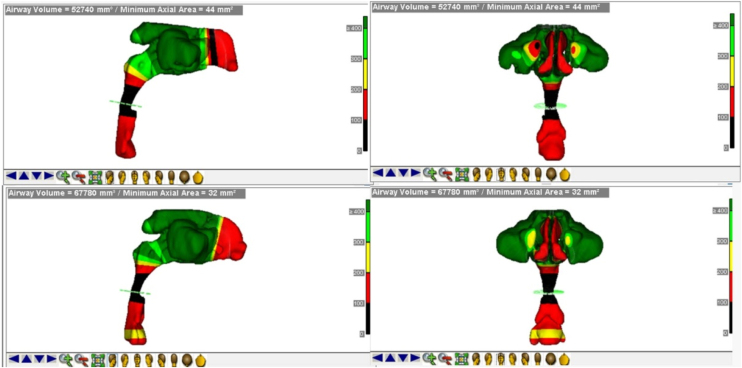
3D mesh demonstrating pre- and post-operative total airway.

When evaluating the hypopharynx, we obtained a mean of 3348 mm^3^ in the preoperative data with a standard deviation of 1881 mm^3^. In the postoperative evaluation, we obtained an average of 4204 mm^3^ with a standard deviation of 2196 mm^3^; the smallest difference was 17 mm^3^ and the largest difference was 3978 mm^3^. We obtained a p-value of 0.0079 after statistical analysis (Table 3), demonstrating that there was a significant difference between the groups (Fig. 3). Evaluating the main airway, we obtained a mean of 3348 mm^3^ with a standard deviation of 1881 mm^3^ in the preoperative data. In the postoperative evaluation, we obtained an average of 74,716 mm^3^ with a deviation of 22,146 mm^3^; the smallest difference was 6 mm^3^ and the largest difference was 77,320 mm^3^. We calculated a p-value < 0.0001 after statistical analysis (Table 3), showing that there was a significant difference between the groups (Fig. 4) and a figure illustrating the 3D mesh of the pre- and post-operative airways of one of the patients (Fig. 5).

Patients who underwent SARME showed an increase in nasal width and an increase in the width of the alar base in the facial soft tissue as well as an increase in the nasolabial angle and in the upper airways.

## Discussion

The main purpose of the study was to evaluate possible alterations in the profile of the facial soft tissue and volumetric alterations of the upper airways in patients submitted to SARME using the transpalatal distractor.

Numerous studies have been published describing the skeletal and dentoalveolar effects of SARME; however, few have considered its effects on the soft tissues of the face or the airways, leading to a limited capacity for evidence of these changes subsequent to maxillary expansion.^11^, ^13^

SARME is an effective and relatively safe technique for widening the atretic maxillae. Its effects are not limited to the maxilla but can extend to circum-maxillary structures as well as various other adjacent structures in the face and skull; in particular, it can also influence the anatomy and physiology of nasal structures. The soft tissues of the face, including the nose, have recently been investigated due to the aesthetic component and also in relation to the stability of the results obtained using expansion.^15^, ^16^, ^17^

The volumetric increase in the nasal cavity occurs due to the enlargement of the maxillary segments that make up the floor of the nasal cavity, which may result in an increase in air flow, consequently improving breathing.^18^

In our study, a significant increase was evaluated in all segments: nasal width, alar base and nasolabial angle; two patients showed divergent data in the soft tissue analysis. There was a decrease of 6° in the nasolabial angle and another 1.5 mm in the alar base, possibly due to the transoperative alar base closure technique.

The first studies, which focused on changes in nasal soft tissues, were performed using measurements from digital photographs before and after SARME, directly on the faces of patients using high precision pachymeters or digital cephalometry. These studies analyzed only changes in width and length. Regarding soft tissue width, Berger et al.,^15^ found an average increase of 2 mm after ERMCA. Our study demonstrated similar results with mean increases of 1.37 mm in alar base width and 1.17 mm in alar width. Both results were statistically significant.

Kim et al.^8^ conducted a study of 23 patients (10 men and 13 women) diagnosed with maxillary bone transverse discrepancy or arch length discrepancy, who underwent Rapid Maxillary Expansion (RME) using the rapid maxilla expander manufactured by the brand Dentaurum (Dentaurum Group, Ispringgen, Germany®). As a result of this study, a significant transverse expansion was noted in most points marked in the soft tissue, which corroborates our results.

Fastuca et al.^6^ carried out a randomized clinical trial with the objective of evaluating three-dimensional changes in the soft tissue of patients in the growth phase after rapid maxillary expansion. The scenario and sample population consisted of a treatment group of 17 patients and a control group of the same size. All patients in the treatment group underwent maxillary expansion surgery using a HASS-type expander. The results showed a difference between the groups in the nasal area; nasal width significantly increased by 1.98 mm in the treatment group compared to the control group. The difference in the total volume of the nose was significantly increased in the treatment group compared to the control group, which was mainly related to a significant increase in the volume of the nasal dorsum.

Baysal et al.^3^ published a randomized clinical trial in which they evaluated changes in the alar base region of a treatment group and control group six months after RME surgery; they came to the conclusion that there was an increase of approximately 1 mm.

In this study, we obtained an average difference of 1.37 mm in nasal width and 1.7 mm in the alar base. The increase in the nasolabial angle, with an average of 5.1°, demonstrated that there are changes in the soft tissue in the anterior region of the face. Currently, with the increasing popularity of computer-assisted surgical planning, the quantification and prediction of soft tissue changes has become an essential component of these software programs.

Postoperative CT scans, with a period longer than four months, were selected. According to Nada et al., every surgical procedure causes post-surgical oedema and, it generally takes at least four to six months to eliminate this effect.^19^

Nasal width, alar base width and nasolabial angle were chosen as soft tissue landmarks because they are highly reproducible and easier to understand than other soft tissue landmarks. Furthermore, it would be somewhat difficult to calibrate other soft tissue landmarks and discuss the validity of these landmarks.

One of the problems of this study was the small sample size. However, this did not influence the conclusions as the data were robust and similar to the literature (Aras et al. (11-patients); Günbay et al. 2008 (10-patients); Rubim de Assis et al. (13-patients)).^2^, ^20^

The surgical procedure itself has been invasive and it has removed, even partially, the soft tissues inserted in this region, especially around of the piriform aperture. Such fact, by itself, could lead to alteration in alar base. In order to minimize possible alterations, the sutures in these structures have been performed, which seemed to positively contribute for decreasing such alterations, since this study’s data revealed a significant increase of the alar base width between the pre-operative and the 4-month postoperative periods. Nary Filho et al.,^21^ considered that the SAME procedures without alar base suture would lead to an invariable increase of the nasal ala. This study’s data revealed that even when the alar base suture was executed along with the V–Y suture, there was a statistically significant increase of the alar base. It has been believed that such fact would result from the enlargement of the osseous portion of the lateral wall base of the piriform aperture, where it is inserted, the soft tissues related to it.^21^

With the evolution of CT-scans, readily available and widely used in dentistry, we have an ideal tool to assess the upper airway, soft tissues and tissue relationships.^22^, ^23^

Regarding the airways, we obtained an increase in the entire respiratory airway, nasopharynx, oropharynx, and hypopharynx. The greatest gains were obtained in the nasopharynx, with the highest average of 48,094 mm^3^.

El et al., in a study of 70 patients evaluating pre- and postoperative CT-scans, showed that SARME creates a significant increase in the nasopharynx but with no alteration in the oropharynx region, which is in contrast to our results, as we demonstrated statistically significant differences in all of the studied variables.^24^ We believe that these differences in results occurred because the author performed their study with linear and angular measurements, while ours three-dimensionally assessed airway volume.

## Conclusion

The results showed that there was a statistically significant increase in all segments of the upper airway, as well as an increase in changes with regard to the repercussion of the soft tissues of the face, width of the alar base, alar width and length and tissue at the height of the nose.

## Funding

This research did not receive any specific grant from funding.

## Conflicts of interest

The authors declare no conflicts of interest.
